# Elevated Growth Differentiation Factor-15 Is a Biomarker of Sarcopenia in Older Adults

**DOI:** 10.1093/geroni/igaa057.433

**Published:** 2020-12-16

**Authors:** Miji Kim, Chang Won Won

**Affiliations:** 1 College of Medicine, Kyung Hee University, Seoul, Republic of Korea; 2 Kyung Hee University Medical Center, Seoul, Korea, Seoul, Republic of Korea

## Abstract

Growth differentiation factor 15 (GDF-15) is associated with disease progression, mitochondrial dysfunction and mortality. Elevated GDF-15 level was recently reported to be associated with poorer physical performance in healthy community-dwelling adults. However, the relationship between serum GDF-15 concentration and sarcopenia in community-dwelling older adults has not been well characterized. We analyzed 929 participants (mean age 75.9±8.9 years, 48.0% men) from the Korean Frailty and Aging Cohort Study who underwent assessment of serum GDF-15 concentration and sarcopenia. Participants with an estimated glomerular filtration rate <60 ml/min/1.73 m2 were excluded from this analysis. Sarcopenia status was determined as per the Asian Working Group for Sarcopenia (AWGS) 2019 guidelines. As per the AWGS 2019 algorithm, 154 (16.6%) participants in the study population were classified as having sarcopenia. Median serum GDF-15 concentration was elevated in the sarcopenic group vs. the non-sarcopenic group (920 vs. 793 pg/ml, p<0.001). In the multivariate analysis adjusted for potential confounders, the highest GDF-15 tertile (≥1245 pg/ml) was associated with a higher risk of sarcopenia vs. the lowest tertile (<885 pg/ml) (odds ratio [OR] = 1.95, 95% confidence interval [CI] 1.15–3.31). This association remained unchanged (OR = 1.90, 95% CI 1.14–3.23) after further adjustment for potential biomarkers (myostatin, dehydroepiandrosterone, and insulin-like growth factor-1). The OR per unit increase in log-transformed GDF-15 concentration was 3.59 (95% CI 1.21–10.70). To conclude, our results suggest that higher circulating GDF-15 concentration was independently associated with a greater risk of sarcopenia in community-dwelling older adults. Serum GDF-15 concentration can be a promising biomarker for sarcopenia

